# Pyridine Controlled Tin Perovskite Crystallization

**DOI:** 10.1021/acsenergylett.2c01749

**Published:** 2022-09-01

**Authors:** Giuseppe Nasti, Mahmoud Hussein Aldamasy, Marion Alwine Flatken, Pellegrino Musto, Piotr Matczak, André Dallmann, Armin Hoell, Artem Musiienko, Hannes Hempel, Ece Aktas, Diego Di Girolamo, Jorge Pascual, Guixiang Li, Meng Li, Lucia Vittoria Mercaldo, Paola Delli Veneri, Antonio Abate

**Affiliations:** † Department of Chemical Materials and Production Engineering, University of Naples Federico II, Piazzale Vincenzo Tecchio 80, 80125 Naples, Italy; ‡ Department of Novel Materials and Interfaces for Photovoltaic Solar Cells, Helmholtz-Zentrum Berlin für Materialien und Energie GmbH, Hahn-Meitner-Platz 1, 14109 Berlin, Germany; § Egyptian Petroleum Research Institute, 4441312 Cairo, Egypt; ∥ National Research Council of Italy Institute for Polymers Composites and Biomaterials, Via Campi Flegrei 34, 80078 Pozzuoli (NA), Italy; ⊥ Faculty of Chemistry, University of Łódź́́́, 90-149 Lodz, Poland; ¶ Humboldt Universität zu Berlin, Institut für Chemie, Brook-Taylor-Str. 2, 12489 Berlin, Germany; ⊗ Key Lab for Special Functional Materials of Ministry of Education, National and Local Joint Engineering Research Center for High-Efficiency Display and Lighting Technology, School of Materials Science and Engineering, Collaborative Innovation Center of Nano Functional Materials and Applications, Henan University, Kaifeng 475004 China; ∇ Italian National Agency for New Technologies, Energy and Sustainable Economic Development (ENEA) - Portici Research Center, Piazzale E. Fermi, 80055 Portici (NA), Italy

## Abstract

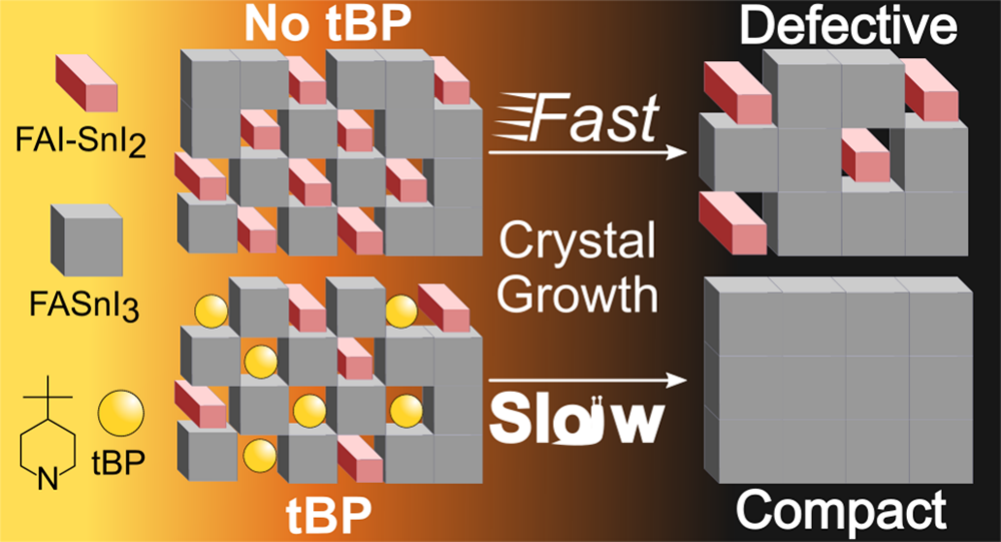

Controlling the crystallization of perovskite in a thin film is essential in making solar cells. Processing tin-based perovskite films from solution is challenging because of the uncontrollable faster crystallization of tin than the most used lead perovskite. The best performing devices are prepared by depositing perovskite from dimethyl sulfoxide because it slows down the assembly of the tin–iodine network that forms perovskite. However, while dimethyl sulfoxide seems the best solution to control the crystallization, it oxidizes tin during processing. This work demonstrates that 4-(*tert*-butyl) pyridine can replace dimethyl sulfoxide to control the crystallization without oxidizing tin. We show that tin perovskite films deposited from pyridine have a 1 order of magnitude lower defect density, which promotes charge mobility and photovoltaic performance.

Metal halide perovskites are a class of materials with the ambition of becoming the new standard for photovoltaics by offering lower costs, easier manufacturing, and more process flexibility.^1−3^ Lead-based perovskites have already demonstrated, in lab-scale devices, their potential for competing with silicon in terms of efficiency (25.7% to date).^4,5^ However, long-term stability and lead toxicity still represent severe concerns for commercializing.^6−13^ A promising solution is substituting lead with tin due to its much lower environmental toxicity^10^ and theoretical higher efficiency. Lead-free tin halide perovskites (THPs) have lower bandgaps than lead, in the optimal range for the highest possible efficiency for a single-junction photovoltaic device, according to the Shockley–Queisser limit.^14^ Nevertheless, THPs are still far from this limit, with the best-certified device reaching 14.6%.^15,16^ Three main issues need to be solved to improve THP performance: optimize the energy band alignment with the selective extracting layers,^17^ decrease the fast crystallization rate,^18^ and eliminate the undesired oxidation of Sn(II) to Sn(IV).^19^ Dimethyl sulfoxide (DMSO) is the most used solvent for preparing both Pb and Sn perovskite thin films due to its favorable interaction with the perovskite precursors in solution, slowing down the crystallization process. However, Saidaminov et al. and our group described its undesirable oxidizing nature when mixed with iodide salts.^20,21^ For this reason, we recently proposed a new group of solvents as possible DMSO replacements without tin oxidation’s downside.^22^ The selection of new solvents for processing THP is a complicated process as many different factors regarding both the chemical and physical properties of the solvents must be considered, for example, the solubility of the precursors, the vapor pressure of the solvents, and the formation of stable complexes with the organic and inorganic components of the salts.^23−25^ One possible approach is to search for an additive or a cosolvent forming a strong interaction with tin iodide salts. Pyridines, and in particular 4-(*tert*-butyl) pyridine (tBP),^26−29^ are common additives in lead halide perovskites, and can form a stable complex with both Sn(II) and Sn(IV) halides through Sn–N coordination.^30^

In this work, we show that tBP can form stable organo-metal complexes with tin iodide salts in DMSO-free solutions, improving the crystallization process of THP perovskite thin films while avoiding the risk of Sn(II) oxidation to Sn(IV). Detailed precursor solution characterization revealed that the binding ability of tBP to Sn(II) can stabilize the colloidal perovskite nanoparticles, forming a stable intermediate state and thus retarding the crystallization dynamics of the THP. These thin films of higher quality showed higher hole mobility and lower defect density, leading to a photocurrent efficiency (PCE) of 7.3%. This efficiency value is the highest reported for solution-processed DMSO-free THP solar cells. It proves the possibility of controlling the perovskite crystallization without using DMSO, a requirement to remove oxidants during the fabrication process entirely.

As confirmed by previous works, the dissolution of the perovskite precursor salts into the solvents leads to the dynamic formation of different colloidal nanoparticles (NPs).^31,32^ These colloidal NPs are the starting point for the nucleation and growth of perovskite grains during the spin coating process. Their chemical nature and stability are crucial for controlling the quality of the perovskite thin film. In the case of THP, the nucleation and growth of these colloids is a fast process that leads to low-quality morphology and noncomplete conversion of SnI_2_ into the perovskite phase, as depicted in Figure1a, with harmful consequences on the final device performance. During the solution’s spin coating, a vast amount of the solvent moves away from the liquid film due to evaporation, and the colloidal NPs start to form larger and more stable nuclei. The dripping of the antisolvent expels the rest solvent. It induces an immediate rise of the solution concentration above the saturation threshold, causing almost instantaneous nucleation and growth of the THP grains, revealed by the rapid change in color of the perovskite film (Movie S1). The fast kinetics of this process does not allow the diffusion and reconstruction of SnI_2_ clusters toward the perovskite phase leading to the formation of a poor morphology that can be recognized by the opaque finishing of the surface (Figure S1a). To reduce the pace of the crystallization, we used the 4-*tert*-butylpyridine (tBP) as a cosolvent. tBP forms strong and stable intermediate complexes with SnI_2_ (Figure 1b), and it is not entirely removed from the thin liquid film during the spin coating process. The slower crystallization process is evident as the THP film color slowly turns from a semitransparent red-brown color to brown-black in around 20 s after the dripping of the antisolvent, and the finishing of the surface is smooth and reflective (Figure S1b). The annealing step also impacts differently on the two films. The films obtained without the addition of the tBP shows little or no change in appearance during the annealing step, confirming that crystallization was already completed during the spin-coating (Movie S2). On the other hand, the films obtained with the addition of the tBP during the annealing step gradually become darker and less transparent. This behavior suggests that the tBP in the film is firmly bonded and requires additional energy to be extracted from the system.

**Figure 1:**
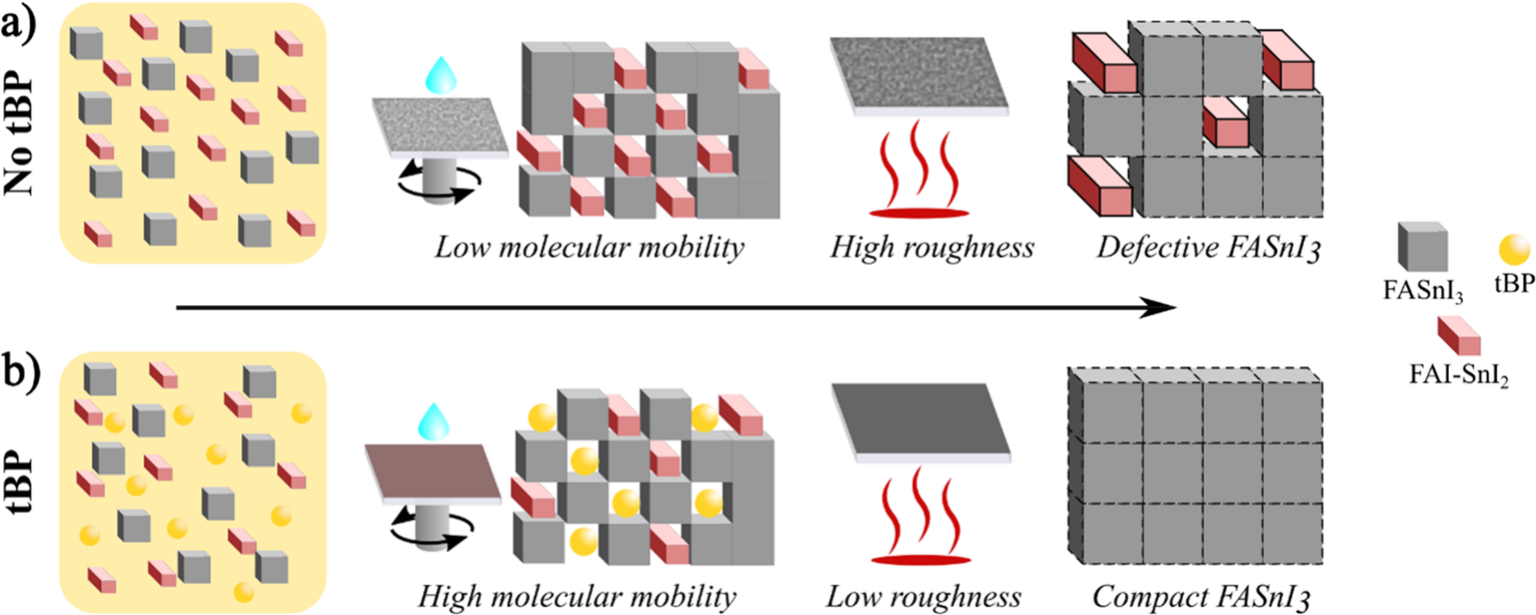
Schematic effect of THP film formation (a) with and (b) without tBP.

Scanning electron microscopy (SEM) imaging confirms the improvement of the THP film morphology (Figure 2a). Without the addition of tBP to the precursor solution, the average grain size is around 200 nm (Figure 2a, left), and the coverage of the substrate is not perfect due to the presence of pinholes. In comparison, the morphology of the THP films obtained with the addition of the tBP has a much larger grain size, between 500 nm and 1 μm (Figure 2a, right), which may be attributed to the slower nucleation and crystallization dynamics in the case of tBP addition. The improvement in the crystallinity is also confirmed by XRD measurements (Figure 2b). In the pattern of tBP-free FASnI3 film, six prominent peaks correspond to the crystallographic planes (100), (102), (200), (122), (222), and (213), indicating an orthorhombic crystal structure. The pattern demonstrates that the tBP-free FASnI3 films consist of random nanocrystals. The (100) and (200) facets were significantly enhanced after the addition of tBP, while the (102), (122), (222), and (213) facets of tBP-free FASnI3 films are absent. The prominence of the (100) and (200) facets can suggest both the formation of a highly oriented crystal film with a preferred orientation of the crystals along these planes or an higher crystallinity of the FASnI3 films.^16^

**Figure 2:**
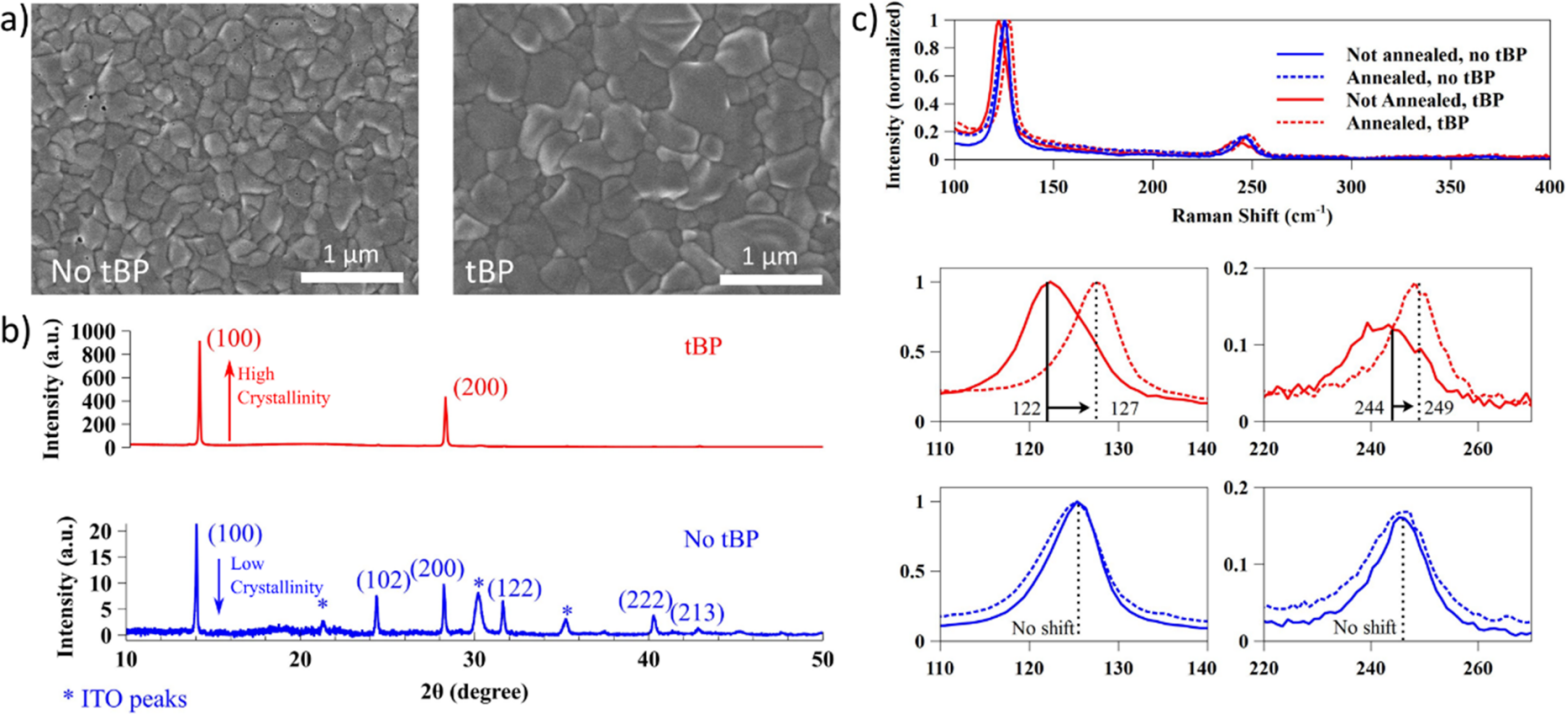
SEM micrographs (a) and XRD plots of THP thin film obtained with and without tBP (b). Raman spectra show the effect of tBP on the vibrational modes of THP and the clear redshift of the low Raman shift peaks before and after annealing (c).

Raman spectroscopy was used to further investigate the effect of the tBP during the annealing step. In Figure 1 and Figure 2c, Raman spectra are reported for THP thin film recorded before and after the thermal annealing of the samples for both the control solution and the solution diluted with tBP. An intense peak centered in the 120–128 cm^–1^ range is present in all spectra. This feature is associated with reticular cage modes and originates from the concerted stretching vibrations of the Sn–I bonds in the crystal lattice.^33,34^ It has been demonstrated that this peak is a diagnostic signature of structural order. It has been proposed to monitor the quality and homogeneity of the deposited layers and investigate fundamental properties such as film thickness, defect density, composition, and stability.^35^ A different band in the 240–248 cm^–1^ range is due to the methylammonium cation, specifically, to a torsional mode of the fragment within the cage structure. Because of the nature of this vibration, the band is broader than that at 120–130 cm^–1^. Its shape and full width at half height (fwhh) are susceptible to interaction with the surrounding environment: the presence of structural defects makes the environment inhomogeneous. It produces a band widening and a multicomponent structure. Because of these characteristics, this feature has been proposed as a marker of the orientational disorder of the material.^34^

Compared to the Raman spectra of related materials (Pb halide perovskites),^34,35^ the present spectra are remarkably narrower and better resolved. A typical lead iodide system displays a multicomponent profile centered at 120 cm^–1^ with an fwhh of 50 cm^–1^. In contrast, in the present case, the single peak at 120–130 cm^–1^ is 1 order of magnitude narrower (fwhh = 5–6 cm^–1^). Analogously, the same lead-based system exhibits a four-component band at 250 cm^–1^ with an fwhh of around 70 cm^–1^, whereas the present spectra show a single Gaussian at 240–248 cm^–1^ with an fwhh of 9–11 cm^–1^. These results indicate a highly ordered structure of the crystals with a negligible concentration of degradation-induced defects. Inspection of Figure 2c reveals that for the control film (blue lines), the position of both the peaks at 125 and 246 cm^–1^ does not change before and after the annealing, suggesting that the crystallization of the THP ends during the spin coating phase and with the antisolvent dripping. During the annealing step, the crystals do not undergo significant structural changes. Conversely, for THP obtained using tBP, after annealing, the peak centered initially at 121 cm^–1^ shifts toward a higher frequency by 6 cm^–1^ and becomes considerably narrower (fwhh goes from 9 to 6 cm^–1^). Analogous effects are observed for the torsional mode, whose position moves from 244 to 249 cm^–1^, while the fwhh decreases by 9 cm^–1^ (from 18 to 9 cm^–1^). These observations suggest that the annealing process in the presence of TPB induces a (further) significant reordering of the lattice structure.

Quantum chemical calculations were carried out to provide a theoretical description of SnI_2_ complexation with tBP, 1,3-dimethyl-2-imidazolidinone (DMI), dimethylformamide (DMF), and DMSO. A series of SnI_2_:solvent (solvent = tBP, DMI, DMF, DMSO) complexes with 1:1 and 1:2 stoichiometry were embedded in a bulk solvent environment (DMF). As shown in Figure 3a (Table S1), the geometrical structure and energetics of these complexes were characterized by their Sn–solvent bond length (*d*), complexation energy (*E*_complex_) and SnI_2_–solvent pairwise interaction energy (*E*_int_). The values of *E*_complex_ indicate that the complexation of SnI_2_ with tBP is more energetically favorable than the formation of the corresponding complexes with DMI, DMF, and DMSO. The most exoenergetic effect of complexation is observed for SnI_2_:2tBP, whose tBP molecules occupy two equatorial positions. Interestingly, the presence of a second tBP molecule in this complex strengthens each of its two SnI_2_–tBP interactions, as evidenced by *E*_int_. By contrast, the preferred isomers of SnI_2_:2DMI, SnI_2_:2DMF and SnI_2_:2DMSO show their solvent molecules sitting at the axial positions, thus the significant elongation of their Sn–O bond length and, consequently, the weakening of their SnI_2_–solvent interactions.

**Figure 3:**
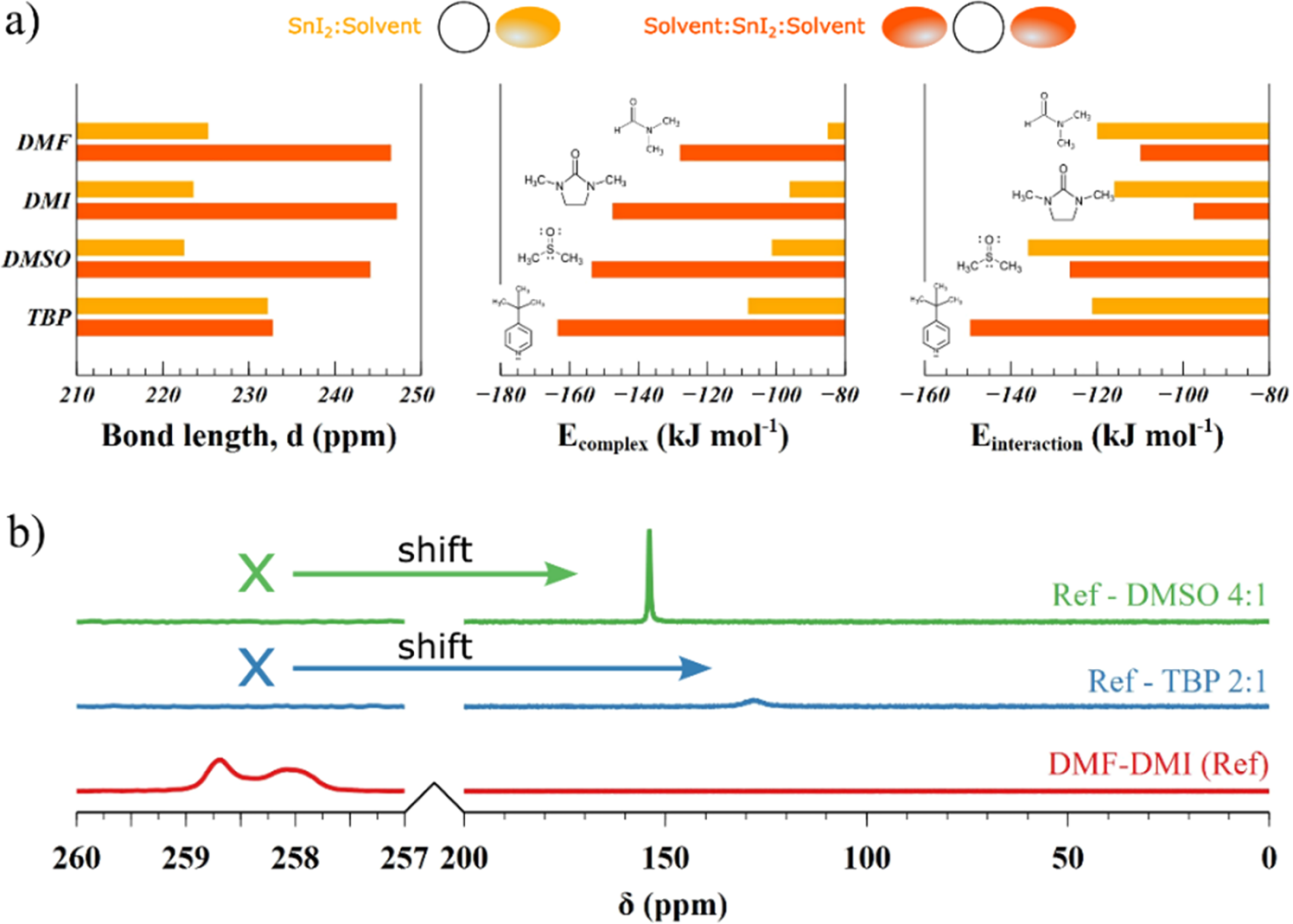
Geometrical (*d*, in ppm) and energetic (*E*_complex_, *E*_int_, in kJ mol^–1^) parameters calculated for the 1:1 and 1:2 complexes in their preferred isomers in DMF solution. (a) ^119^Sn-NMR signals measured for THP precursors in a DMF–DMI 6:1 solution and with the addition of tBP and DMSO as cosolvents, respectively, at 2:1 and 4:1 volume ratios. All solutions were measured at 0.9 M concentration (b).

To further support this theoretical finding, we performed liquid ^119^Sn-NMR to compare the solvation state of tin in different solvent compositions. In Figure 3b, it is shown that for a DMF–DMI 6:1 solution of the perovskite precursors, two partially superimposed peaks can be found at 258.0 and 258.7 ppm. With the addition of both DMSO and tBP, these peaks disappear and are substituted by a single peak for shorter chemical shifts, confirming the much more robust and stable complexing ability of these two molecules compared to DMF and DMI. The close chemical shift positions of DMSO and tBP ensure their similar electron density donation capability to Sn(II).

To demonstrate the effect of the tBP addition on the charge transport, we characterized FASnI_3_ thin films by 4-probe conductivity and the Hall effect with an AC magnetic field. The tBP sample showed much lower conductivity of 3.3 × 10^–3^ Ω^–1^ cm^–1^ compared to the model without tBP addition, which had a higher conductivity value of 8 × 10^–2^ Ω^–1^ cm^–1^ (Figure 4a,b). Such a conductivity decrease in the tBP sample can be due to good crystallinity and a lower concentration of defects. On the other hand, the reduction in conductivity can be affected by hole mobility reduction. Therefore, we performed Hall effect measurements to find carrier concentrations and mobilities directly. On the contrary, the Hall effect measurements revealed a two times larger free hole mobility in samples with tBP addition (0.083 cm^2^ V^–1^ s^–1^) compared to the reference sample with a hole mobility of 0.04 cm^2^ V^–1^ s^–1^ (Figure 4d). In addition, tBP samples demonstrated a ten times lower free hole density in tBP samples, 1.1 × 10^18^ cm^–3^, compared to the reference sample, 1.9 × 10^19^ cm^–3^ (Figure 4c). These results highlight the beneficial effect of tBP addition on charge transport, crystallinity, and defect formation. In particular, the decrease of acceptor defects concentration will raise the radiative lifetime of electrons from 0.1 to 2.3 ns in the tBP sample calculated according to hole concentration, Figure 4c. The concentration, mobility, and lifetime results agree, taking into account the wide range of values measured by other groups, with previous studies.^36^ Measurements of transient photoluminescence decay for THP film with and without tBP directly supported the charge carrier lifetime that increased from 0.4 ns without tBP to 0.6 ns with tBP (Figure 4e).

**Figure 4:**
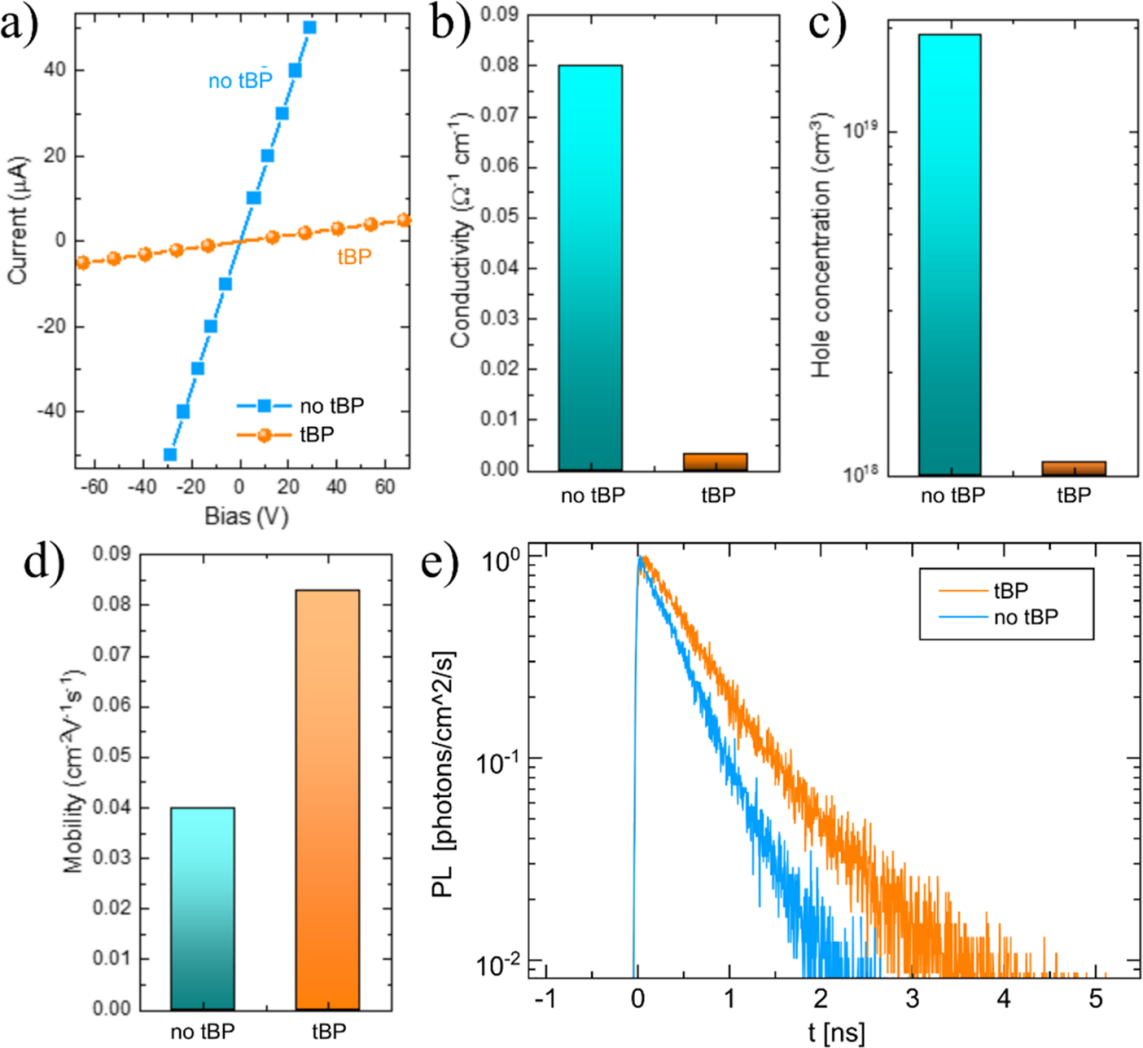
Current-bias measurements of reference and tBP FASnI3 thin films; (a) 4-probe conductivity; (b) Hall concentration probed with AC magnetic field; (c) Hall mobility probed with AC magnetic field; (d) electron radiative lifetime calculated from Hall concentration; (e) experimental photoexcited holes density and theoretical simulation of holes and electrons concentration as a function of generation rate.

We prepared p-i-n devices with the structure ITO/PEDOT/THP/C60/BCP/Ag to prove these films’ photovoltaic performances. Due to the presence of pinholes and the poor morphology, all the devices obtained without adding tBP showed efficiencies below 2%, with poor reproducibility, and are therefore not reported here. The tBP cosolvent, by slowing down the crystallization process, made it possible to access a much wider processing window, increasing both the efficiency and the reproducibility of the devices. Figure 5a,b shows respectively the external quantum efficiency (EQE) and the current density–voltage (*J–V*) characteristics of the best device, showing an open-circuit voltage (*V*_OC_) of 548 mV; a short-circuit current (*J*_SC_) of 18.6 mA/cm^2^ (value confirmed by the integrated current density calculated from the EQE), a fill factor (FF) of 71.7%, and a 7.3% power conversion efficiency (PCE). The *V*_OC_ and FF showed a remarkable statistical distribution with most devices, respectively above 500 mV and 60%, with a slightly larger dispersion of values for the *J*_SC_ and the PCE.

**Figure 5:**
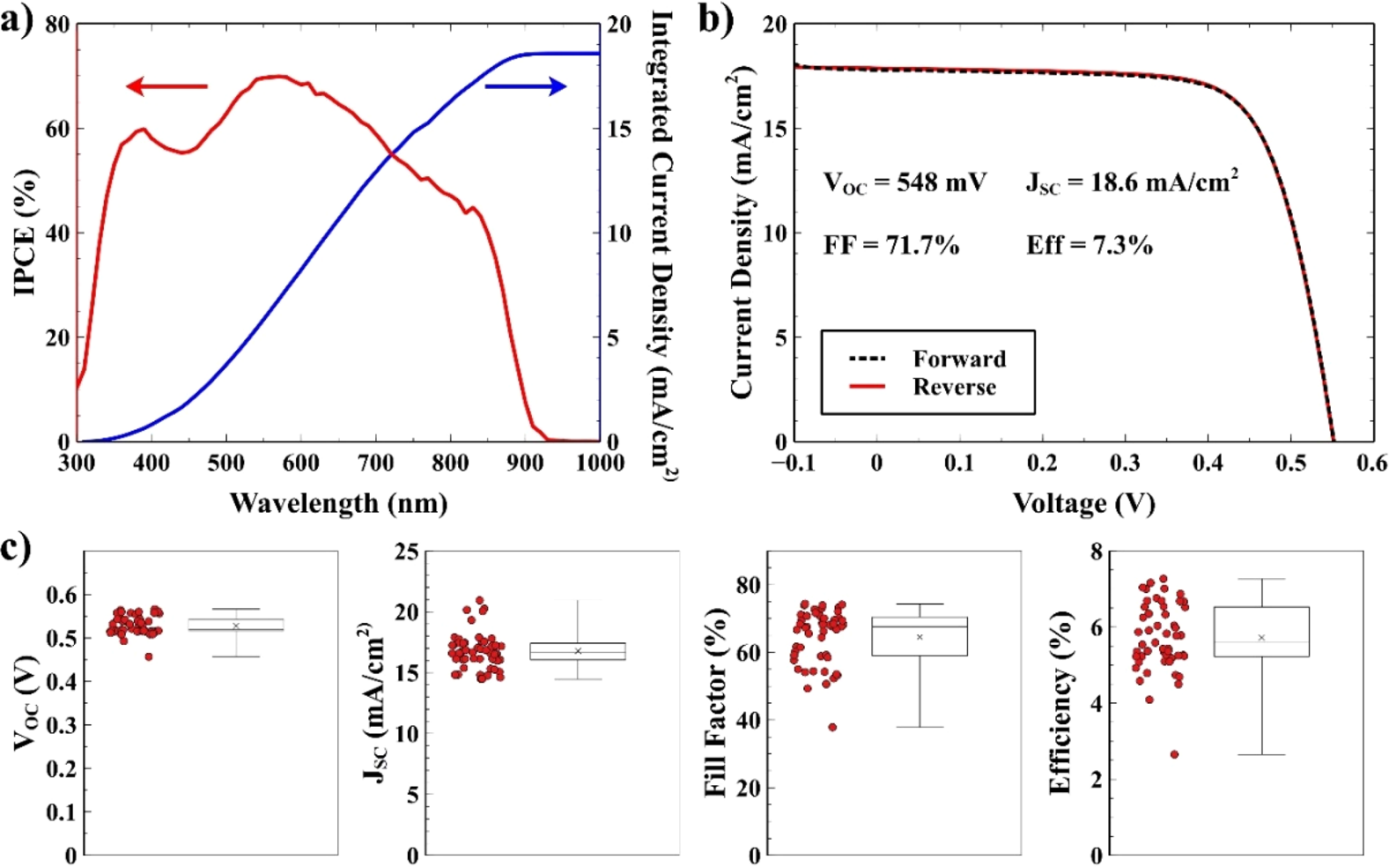
EQE spectra (a) and *J*–*V* characteristics (b) of devices obtained by adding tBP at the beginning and end of the preparation of the perovskite solution.

To summarize, a stable complex between tBP and the tin halide controls the kinetics of the crystal’s nucleation and growth, leading to a pinhole-free morphology with vastly increased crystal grain size. The detailed solution, film characterization, and numerical simulations described the interactions between tBP and THP critically influenced the crystallization. tBP significantly increased the mobility of hole charge carriers by improving the morphology, reducing the defects density and self-doping. A reproducible process was demonstrated to manufacture efficient Pb-free DMSO-free devices, with the best device showing efficiency of 7.3%.

## Supporting Information Available

Experimental materials and methods, small-angle X-ray scattering analysis, theoretical calculations, Hall effect (PDF)
Movie S1: film obtained with the addition of the tBP (MP4)
Movie S2: film obtained without the addition of the tBP (MP4)
