# Supplementary material for: Pyridine Controlled Tin Perovskite Crystallization
Source: ACS Energy Lett. 2022 Sep 1;7(10):3197–203. doi: 10.1021/acsenergylett.2c01749 (PMC9578040; doi:10.1021/acsenergylett.2c01749)
Supplement: Supplementary file 1 — nz2c01749_si_001.pdf [file nz2c01749_si_001.pdf]

Supporting Information
Pyridine Controlled Tin Perovskite Crystallization

Giuseppe Nasti[a,†,*], Mahmoud Hussein Aldamasy[b,c,*], Marion Alwine Flatken[b], Pellegrino Musto[d], Piotr Matczak[e], André Dallmann[f], Armin Hoell[b], Artem Musiienko[b], Hannes Hempel[b], Ece Aktas[a], Diego Di Girolamo[a], Jorge Pascual[b], Guixiang Li[b], Meng Li[g], Lucia Vittoria Mercaldo[h], Paola Delli Veneri[h], Antonio Abate[a,b,†]

a) Department of Chemical Materials and Production Engineering, University of Naples Federico II, Piazzale Vincenzo Tecchio 80, 80125 Naples, Italy

b) Department of Novel Materials and Interfaces for Photovoltaic Solar Cells, Helmholtz-Zentrum Berlin für Materialien und Energie GmbH, Hahn-Meitner-Platz 1, 14109 Berlin, Germany

c) Egyptian Petroleum Research Institute, 4441312 Cairo, Egypt.

d) National Research Council of Italy Institute for Polymers Composites and Biomaterials, Via Campi Flegrei 34, 80078 Pozzuoli (NA), Italy

e) Faculty of Chemistry, University of Łódź, 90-149 Lodz, Poland

f) Humboldt Universität zu Berlin, Institut für Chemie, Brook-Taylor-Str. 2, 12489 Berlin, Germany

g) Key Lab for Special Functional Materials of Ministry of Education, National and Local Joint Engineering Research Center for High-Efficiency Display and Lighting Technology, School of Materials Science and Engineering, Collaborative Innovation Center of Nano Functional Materials and Applications, Henan University, Kaifeng 475004 China

h) Italian National Agency for New Technologies, Energy and Sustainable Economic Development (ENEA) - Portici Research Center, Piazzale E. Fermi, 80055 Portici (Na), Italy

Corresponding authors:

* These authors contributed equally

† Giuseppe Nasti: giuseppe.nasti@unina.it

‡ Antonio Abate: antonio.abate@unina.it

Experimental Section

Materials

Unless otherwise specified, our study used chemicals as received without further purification. Tin(II) iodide (SnI_2), ethylenediammonium diiodide ($EDAI_2$, >98%), *N*,*N*-dimethylformamide (anhydrous, 99.8%), 1,3-dimethyl-2-imidazolidinone (DMI), 4-(*tert*-butyl) pyridine (98%), cyclohexane (anhydrous 99.5%), ethanol (absolute, EMPLURA), acetone (EMPLURA), Hellmanex, C60 (99.5%), bathocuproine (BCP), and Silver beads III were purchased by Sigma-Aldrich and used as received. Formamidinium iodide (FAI, > 98%) and methylammonium iodide (MAI, 98%) were purchased from Dyenamo. Patterned ITO glass slides (OLED grade, 10 Ohm/sq) were purchased from Automatic Research GmbH. PEDOT:PSS polymer dispersion in toluene (CLEVIOS™ HTL Solar 3) was purchased from Heraeus.

Perovskite solution preparation

A solution of SnI_2 in DMF:DMI at a 6:1 volume ratio was prepared with nominal molarity of 1.2 M. The solution was kept under shaking at ambient temperature overnight to dissolve the tin salts completely. The density was checked to calculate the actual molarity of the solution. In a new vial, FAI powder was scaled, and the proper amount of the tin iodide solution was added to the powder using a calibrated micropipette leading to a FA:Sn molar ration of 0.78:1. The solution was kept under shaking for an hour at ambient temperature for the complete dissolution of FAI. The density of the $FA_{0.78}SnI_3$ solution was measured to calculate the molarity of the solution. In a new vial, MAI and $EDAI_2$ were scaled. The proper amount of the $FA_{0.78}SnI_3$ solution was added to the powder using a calibrated micropipette leading to a FA:MA:$EDAI_2$:Sn molar ratio of 0.78:0.2:0.02:1 molar ratio. The solution was kept under shaking for one hour at ambient temperature to dissolve MAI and $EDAI_2$ completely. The $FA_{0.78}MA_{0.2}EDAI_{0.02}SnI_3$ solution was diluted with tBP in the ratio of 2:1 and kept under shaking for 10 minutes at ambient temperature. For samples obtained by addition of tBP at the beginning of the perovskite solution preparation, the procedure was modified by preparing a 0.9 M nominal solution of SnI_2 instead of the 1.2 M and with a solvent mixture of DMF:DMI:tBP at 6:1:4.5 to consider the solution dilution and the solvents final ratios.

Solar cells fabrication

An inverted structure was chosen for the PSCs fabricated with the following stacking: glass/ITO/PEDOT:PSS/$FA_{0.78}MA_{0.2}EDAI_{0.02}SnI_3$/$C_{60}$/BCP/Ag. Patterned indium tin oxide (ITO) coated glasses were first washed in an ultrasonic thermal bath at 40°C with the following procedure: 15 minutes with a liquid detergent dissolved in deionized water (2% V/V); rinsed in deionized water and sonicated for 5 minutes; rinsed in acetone and then sonicated for 15 minutes; rinsed in ethanol and then sonicated for 15 minutes; dried with a dry air gun to prevent the formation of stains. After this step, the substrates were transferred into a nitrogen-filled glovebox (O_2 < 0.1ppm, H_2O < 0.1ppm, temperature 25°C) for all

the following steps. Before depositing the HTL, the substrates were treated with 10 minutes of N_2 plasma. Diluted Clevious HTL Solar 3 in toluene (1:6 V/V) was deposited using spin coating at 5000 rpm for 30 seconds and then annealed at 150°C for 10 minutes. Using a micropipette, 50 µL of the perovskite solution was deposited on the substrate and spread with a disposable pipette tip, simulating a blade coating technique, due to the low wettability of the liquid on the PEDOT surface, before starting the spin coater at 5000 rpm for 45 seconds. Cyclohexane was used as an antisolvent and released after 15 s from the beginning of the rotation. The perovskite films were thermally annealed for 30 minutes at 100°C. The ETL and the Silver electrode were deposited using a thermal evaporation chamber at a high vacuum ($<10^{-6}$ bar). The thickness of the three layers, C_{60}, BCP and Ag, were 32, 10 and 120 nm, respectively.

Photovoltaic characterization

The multimodal Arkeo machine from Cicci Research Srl was used to conduct the characterization in the glovebox. The device's current density-voltage (*J-V*) characteristics were tested using a 12 LED solar simulator with a calibrated optical power density of 100 mW/cm^2. PSC *J-V* characteristics were measured every 10 mV with a sample rate of 100 mV/s. The active area of all samples was 0.1 cm^2. Maximum Power Point Tracking (MPPT) was performed using the same light intensity. External Quantum Efficiencies (EQE) were measured using a neon white light source with a "name" monochromator. The monochromatic light intensity was calibrated using a Hamamatsu S1337 Si calibrated photodiode.

Raman analysis

The Raman spectra were collected by a confocal Raman spectrometer (Horiba-JobinYvon Mod. Labspec Aramis) operating with a diode-pumped laser source emitting at 632.8 nm. The 180° back-scattered radiation, passed through a notch filter to eliminate the elastic component, was collected by an Olympus metallurgical objective (MPlan 50x, long-working distance, NA = 0.75) with confocal and slit apertures both set to 400 µm. A 1200 grooves/mm grating was used to resolve the frequency components. Five consecutive scans with an exposure time of 10 s were averaged to improve the Signal-to-noise ratio. The radiation was focused onto a CCD detector (Synapse Mod. 354308) cooled at -70°C by a Peltier module. It was operated in the Raman-shift range of 50 – 1000 cm-1. The laser power measured at the output of the objective was 65.2 µW, which resulted in 740 W/cm2 in terms of power density. All measurements were carried out with a 25% nominal attenuation (actual value 210 W/cm2) to avoid any damage to the crystal surface caused by irradiation [R3]. The spectra were calibrated using a monocrystalline silicon wafer as a reference (primary Raman mode at 520 cm−1).

NMR analysis

The spectra were all acquired on a Bruker AVII 400 MHz equipped with a room-temperature TBO probe head. Typically, a sweep width of 504.3 ppm was used, and 64k points were acquired, resulting in a total acquisition time of 435.81 ms. The centre frequency had to be adjusted from sample to sample to

detect the desired signal; therefore, on new samples, a full scan of the possible shift range was acquired (1000 to -3000ppm). We used a 30° pulse to reduce the recycle delay to 2s. The number of scans thus ranged from 128 for very concentrated samples to 16k scans for very dilute samples.

XRD analysis

Bruker AXS D8 ADVANCE X-ray diffractometer (multipurpose diffractometer) and Bruker AXS DIFFRAC.SUITE software was utilized for the measurements. Samples were stored in a nitrogen atmosphere using half-spherical domes to prevent air oxidation. Patterns were collected with a step size of 0.01° and a step duration of 2 seconds.

SAXS analysis

SAXS measurements were performed using synchrotron radiation at the four-crystal monochromator (FCM) beamline of the Physikalisch-Technische Bundesanstalt (PTB), the German Metrology Institute,[40] at the BESSY II synchrotron (Helmholtz-Zentrum Berlin für Materialien und Energie (HZB), Berlin, Germany). The beamline was combined with the HZB ASAXS instrument.[41] The incoming photon flux was monitored with an 8 μm thin diode operating in transmission mode. The transmitted beam was measured with a photodiode inside the beamstop in front of the scattering detector. The SAXS patterns were recorded with a windowless 2D X-ray hybrid pixel detector (Pilatus 1M, Dectris Ltd, Switzerland)[42] at two different distances (~0.8 m and ~3.8 m) at two photon energies of 10 keV and 8 keV, respectively. Thus, a q-range from about 0.05 nm-1 to 8.5 nm-1 was covered. Due to the low transmittance of the tin-containing precursor solutions, especially thin (0.1 mm), rectangular borosilicate cuvettes (with a wall thickness of 0.1 mm) purchased from CM Scientific, UK were used.

The magnitude of the scattering vector was calibrated using the d-spacing of a silver behenate standard sample. To calibrate all curves into differential scattering cross-sections, the scattering of glassy carbon was measured together with each sample sequence. The collected two-dimensional raw scattering data patterns were corrected for possible variations in the incoming photon flux, sample transmission, scattering background and geometrical effects by spherical projection. The scattering background from beamline components was measured within each sample sequence using an empty capillary. The scattering images were azimuthally averaged to scattering curves around the beam center.

Hall Effect and Transient PhotoLuminescence

We used AC magnetic field with a lock-in amplifier to enhance a low Hall effect signal due to low mobility and high conductivity values. 4-probe Hall effect and mobility were measured on encapsulated $FASnI_3$ samples in He environment at room temperature. We used 0.6 T magnetic field amplitude and a frequency of 100 Hz. We do not observe any significant resistivity variation before ($2.5 \cdot 10^4$ Ω) and after ($2.49 \cdot 10^4$ Ω) measurements; thus, the ionic conductivity can be neglected. A time-correlated single photon counting system measured TRPL. Photogeneration was achieved with laser pulses with a wavelength of 515nm, a pulse length of 250 fs, a repletion rate of 125kHz and a spot size of ~1 mm. The photoluminescence was selected by a 600 nm long pass and a 550 nm long pass.

Computational details

The geometrical structure of all studied complexes and their molecular constituents (the separated molecules of SnI_2, tBP, DMI, DMF, and DMSO) was fully optimized at the DFT level of theory. The BP-D dispersion-corrected density functional[37–39] was combined with the aug-cc-pVTZ-PP[40] (for the atoms of SnI_2) and aug-cc-pVTZ[41] (for the remaining atoms) basis sets. Bulk solvent effects on the geometrical structure and energy were evaluated using the SMD implicit solvation model[42]. Harmonic vibrational frequency calculations were performed at the BP-D/aug-cc-pVTZ(-PP) level to verify that the optimized geometries corresponded to (local) minima on the $3N$-6 dimensional potential energy surface. The formation of each complex was characterized by its complexation energy (E_{complex}):

$$E_{\text{complex}} = E_{\text{int,tot}} + E_{\text{def}} + \Delta\text{ZPVE},$$

where $E_{\text{int,tot}}$ is the total interaction between the molecular fragments of the complex, E_{def} is the energy consumed by the geometrical deformation from the separated molecules to the corresponding molecular fragments of the complex, and ΔZPVE denotes the difference between the zero-point vibrational energies of the complex and its separated molecular constituents. The E_{complex} energy was calculated at the BP-D/aug-cc-pVTZ(-PP) level. Additionally, the interaction energy (E_{int}) for each SnI_2-solvent pair in the complexes was also obtained from single-point SCS-MP2/aug-cc-pVTZ(-PP)[43] calculations. All calculations were carried out using the Gaussian 16 C.01 program[44].

Morphology

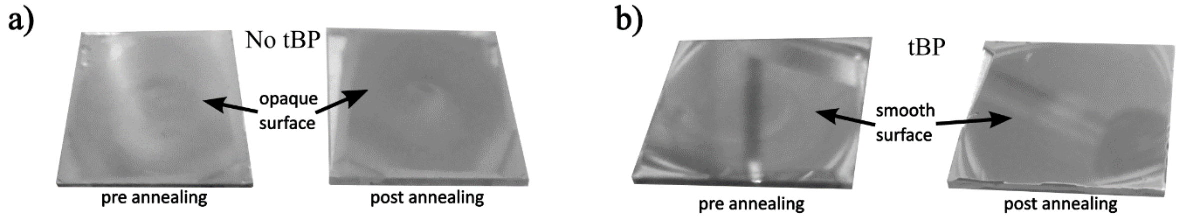

Figure S1: opaque and smooth morphologies obtained with a) and without b) tBP.

Small Angle X-Ray Scattering Analysis

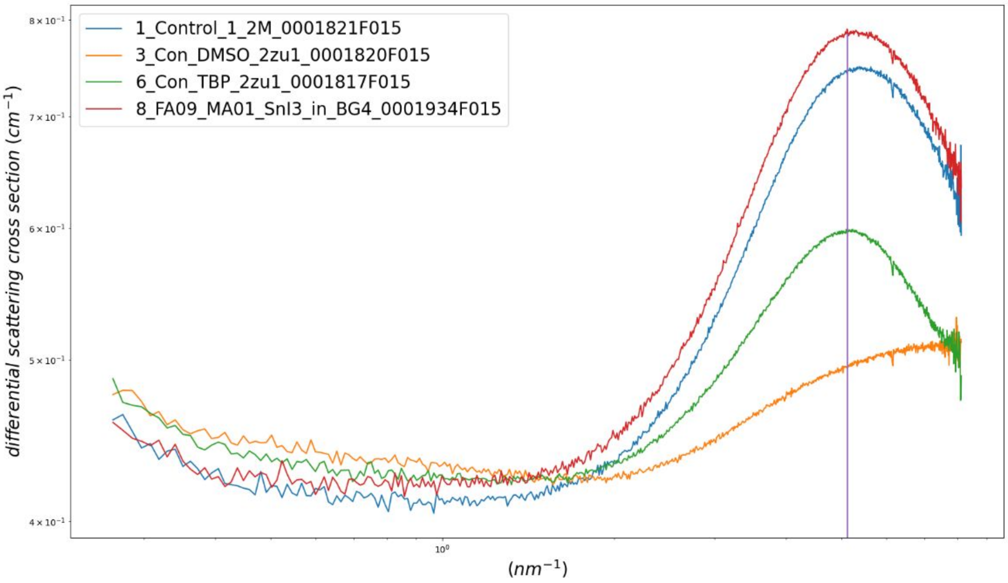

Figure S2: Small Angle X-Ray Scattering curves of four distinguished solution samples measured at room temperature are shown. THP (8, red) is shown with respect to the control solution 1, blue. The second pair are THP in TBP and DMSO solutions (curves 6, green and 3, dark yellow). A vertical line is shown as guide for the eyes around the maxima of the scattering curves.

Small angle X-ray Scattering was measured at four fresh prepared THP solutions at room temperature. The differential scattering cross sections are shown as a function of the scattering vector, *q*, in fig. 4. The scattering of sample 8, containing TBP is comparable to sample 1, without TBP in the solution. The scattering curves are indicating a pronounced arrangement maxima at q ~ 5.2 nm^{-1} in both cases. This is a spacing distance of around 1.2 nm between the basis construction groups. Therefore, we have no significant changes through the addition of TBP in the solution.

Comparing the samples 6 (with TBP) and 3 (same composition only TBP exchanged by the same amount of DMSO) shows pronounced differences. While these sample has a similar arrangement as the two samples 8 and 1, the DMSO containing sample behaves different. That solution is showing a much weaker ordering effect with smaller distances than 1.2 nm. A maximum could not be found in the accessible q-range up to 8 nm^{-1}.

Theoretical Computations

List of abbreviations

TBP – 4-*tert*-butylpyridine

DMI – 1,3-dimethyl-2-imidazolidinone

DMF – *N*,*N*-dimethylformamide

DMSO – dimethylsulfoxide

Table S1: Geometrical (d, in ppm) and energetic ($E_{complex}$, E_{int}, in kJ mol^{-1}) parameters for the 1:1 and 1:2 complexes in their preferred isomers in DMF solution. E_{int} calculated at the SCS-MP2/aug-cc-pVTZ(-PP) level of theory.

Complex	Parameter		
	d	E_{complex}	E_{int}
SnI_2:TBP	232.2	-108.2	-121.1
SnI_2:DMSO	222.5	-101.3	-136.0
SnI_2:DMI	223.6	-96.1	-116.0
SnI_2:DMF	225.3	-85.1	-120.0
SnI_2:2TBP	232.8	-163.5	-149.5
SnI_2:2DMSO	244.1	-153.6	-126.3
SnI_2:2DMI	247.2	-147.5	-97.5
SnI_2:2DMF	246.5	-128.0	-109.9

The geometrical structure of all studied complexes and their molecular constituents (SnI_2, TBP, DMI, DMF) was fully optimized at the density functional theory (DFT) level of theory. The complexes were formed by one SnI_2 molecule whose Sn center coordinated one or two molecules of a solvent (TBP, DMI or DMF). For the resulting 1:1 and 1:2 complexes, a number of their initial geometries showing various σ-type coordination modes (Sn←N and Sn←O) and different positions of ligands in the coordination polyhedron were optimized to detect their preferred isomers. The Becke-Perdew dispersion-corrected density functional (BP-D) [1],[2],[3], combined with the aug-cc-pVTZ-PP [4] (for the atoms of SnI_2) and aug-cc-pVTZ [5] (for the remaining atoms) basis sets, was used in the optimizations. Previous studies showed that this level of theory is capable of predicting the equilibrium structures and energetics of Sn(II) complexes accurately [6],[7]. The optimizations of complexes were performed both in their gas phase (that is, for the isolated complexes) and in bulk solvent environments (that is, in DMF and DMSO solutions). In the latter case, the 1:1 and 1:2 complexes were embedded in the DMF or DMSO solvent continuum. The solvation model based on density (SMD) [8], which is an

implicit solvation model, was used to evaluate bulk solvent effects on the geometrical structure and energy of complexes. Harmonic vibrational frequency calculations were performed at the BP-D/aug-cc-pVTZ(-PP) level to verify that the optimized geometries corresponded to (local) minima on the $3N$-6 dimensional potential energy surface.

The formation of each complex was characterized by its complexation energy ($E_{complex}$) defined as:

$$E_{complex} = E_{int,tot} + E_{def} + \Delta ZPVE,$$

where $E_{int,tot}$ is the total interaction between the molecular fragments of the complex, E_{def} is the energy consumed by the geometrical deformation from the separated molecules to the corresponding molecular fragments of the complex, and ΔZPVE denotes the difference between the zero-point vibrational energies of the complex and its separated molecular constituents. The $E_{complex}$ energy was calculated at the BP-D/aug-cc-pVTZ(-PP) level. The negative value of $E_{complex}$ indicates that the formation of the complex is energetically favorable. Similarly, the negative value of $E_{int,tot}$ shows undoubtedly an attractive interaction between the moieties and this interaction stabilizes the complex. Additionally, the interaction energy (E_{int}) of each SnI_2-solvent pair in the complexes was also obtained from single-point SCS-MP2/aug-cc-pVTZ(-PP) [9] calculations. Thus, the DFT results could be verified by an advanced wave function theory (WFT) method. For 1:1 complexes, their E_{int} energy is equivalent to $E_{int,tot}$. Core electrons were excluded from the correlation treatment within the SCS-MP2 method. The choice of the SCS variant of MP2 was justified by its good performance in predicting the energetics of Sn(II) coordination complexes [10]. The $E_{int,tot}$ and E_{int} energies were calculated using the basis set of the whole complex. In other words, the counterpoise correction proposed by Boys and Bernardi [11] was employed to remove the basis-set superposition error from the values of $E_{int,tot}$ and E_{int}.

All calculations were carried out using the Gaussian 16 C.01 program [12].

Complexes of 1:1 form

A series of SnI_2:solvent (solvent = TBP, DMI, DMF) complexes with 1:1 stoichiometry were examined at the first stage of the computational part of the study. A set of initial SnI_2:TBP geometries generated for their optimization assumed the σ-complexation of the N-donor of TBP to the Sn center of SnI_2. It is known that the π-complexation of stannylenes with pyridine and its derivatives is significantly weaker than the Sn←N σ-complexation with these ligands [13]. Therefore, only the σ-complexation mode was examined for the SnI_2:TBP complex. As for the SnI_2:DMI and SnI_2:DMF complexes, the sets of their initial geometries included both the Sn←N and Sn←O coordination patterns of σ-complexation. Out of the optimized geometries corresponding to the local energy minima of the three complexes, the lowest-energy structures were selected and their several geometrical and energetic parameters are listed in Table S1. These structures optimized in the presence of bulk DMF solvent are depicted in Figure S1.

From the $E_{complex}$ values presented in Table S1, it can be deduced that the formation of SnI_2:TBP in the DMF or DMSO bulk solvent environment is more energetically favorable than the formation of SnI_2:DMI and SnI_2:DMF in the solutions. The complexation of SnI_2 with a single DMF molecule produces the least exoenergetic effect. The Sn←O coordination bond occurring in the SnI_2:DMI and SnI_2:DMF complexes leads to a greater stabilization than the complexation involving the N-donors of DMI and DMF. The preference for the complexation through the O-donors of DMI and DMF is associated with the greater interaction between the molecular fragments of SnI_2:DMI and SnI_2:DMF, as evidenced in the $E_{int,tot}$ values in Table S1. Moreover, this preference is also manifested in the Sn←O bond lengths (*d* in Table S1) that are shorter than the Sn←N coordination bonds of SnI_2:DMI and SnI_2:DMF. The preference for the Sn←O bond in SnI_2:DMI and SnI_2:DMF is in agreement with the coordination pattern reported for various metal complexes bearing DMI and DMF ligands [14-17]. It is well known that such complexes in solutions and in crystals show their metal centers attached to the O-donors of DMI and DMF.

The geometry of SnI_2 undergoes a slight distortion upon complexation with the molecules of TBP, DMI or DMF. The Sn–I bonds (*d* in Table S1) are elongated and the I–Sn–I angle (*a*) between these bonds usually decreases slightly upon complexation. The two geometrical parameters for the SnI_2 molecule in its optimized geometry are presented in Table S2.

The geometrical and energetic parameters calculated for the complexes in the presence of bulk DMF and DMSO solvents can be compared with the corresponding results obtained for the complexes in their gas phase in order to estimate bulk solvent effects. The comparison of $E_{complex}$ values reveals the pronounced effect of DMF and DMSO environments on the high stabilization of the SnI_2:TBP complex. In the gas phase the formation of SnI_2:DMI turns out to be slightly more energetically favorable than the TBP complexation of SnI_2. Both the DFT and WFT calculations of $E_{int,tot}$ agree that the interaction between SnI_2 and DMI in their gas-phase complex exhibiting the Sn←O coordination is stronger than that observed for SnI_2:TBP in the gas phase.

Table S2. Geometrical and energetic parameters for three 1:1 complexes in DMF solution. Values of the parameters for the complexes in DMSO solution and in gas phase are given in parentheses and square brackets, respectively. [a]

Parameter	Complex/coordination				
	SnI_2:TBP	SnI_2:DMI		SnI_2:DMF	
	Sn←N	Sn←O	Sn←N	Sn←O	Sn←N
d(Sn←donor)	232.2<br>(232.1)<br>[239.3]	223.6<br>(223.6)<br>[229.0]	251.0<br>(250.9)<br>[257.1]	225.3<br>(225.2)<br>[232.6]	276.6<br>(265.8)<br>[280.5]
d(Sn–I)	288.3	289.5	286.5	288.8	283.9;284.5

	(288.4)<br>[282.7]	(289.6)<br>[284.5]	(286.5)<br>[282.3]	(289.0)<br>[283.6]	(283.8;285.6)<br>[279.8;280.4]
a(I–Sn–I)	95.7<br>(95.6)<br>100.7	95.3<br>(95.1)<br>[98.9]	96.7<br>(96.2)<br>[99.7]	95.1<br>(95.0)<br>[97.4]	97.0<br>(96.8)<br>[99.5]
$E_{complex}$	-108.2<br>(-109.4)<br>[-108.7]	-96.1<br>(-96.3)<br>[-110.5]	-87.0<br>(-88.2)<br>[-96.3]	-85.1<br>(-85.0)<br>[-92.5]	-49.8<br>(-50.3)<br>[-57.4]
$E_{int,tot}$ [b]	-131.9<br>(-142.3)<br>[-120.9]	-132.0<br>(-140.4)<br>[-133.6]	-113.7<br>(-120.7)<br>[-115.2]	-124.0<br>(-131.2)<br>[-108.0]	-72.5<br>(-82.9)<br>[-71.5]
$E_{int,tot}$ [c]	-121.1<br>(-127.9)<br>[-109.1]	-116.0<br>(-120.9)<br>[-120.4]	-89.5<br>(-93.9)<br>[-94.6]	-120.0<br>(-124.2)<br>[-106.5]	-52.2<br>(-65.1)<br>[-52.7]

[a] Distances (*d*) are given in pm, angles (*a*) in degrees, energies ($E_{complex}$, $E_{int,tot}$) in kJ mol^{-1}.

[b] Energies calculated at the BP-D/aug-cc-pVTZ(-PP) level of theory.

[c] Energies calculated at the SCS-MP2/aug-cc-pVTZ(-PP) level of theory.

Table S3. Geometrical parameters for SnI_2 in DMF solution. Values of the parameters for SnI_2 in DMSO solution and in gas phase are given in parentheses and square brackets, respectively. [a]

Parameter	Value
d(Sn–I)	281.4<br>(281.4)<br>[276.2]
a(I–Sn–I)	97.5<br>(97.3)<br>[100.4]

[a] Distance (*d*) is given in pm, angle (*a*) is expressed in degrees.

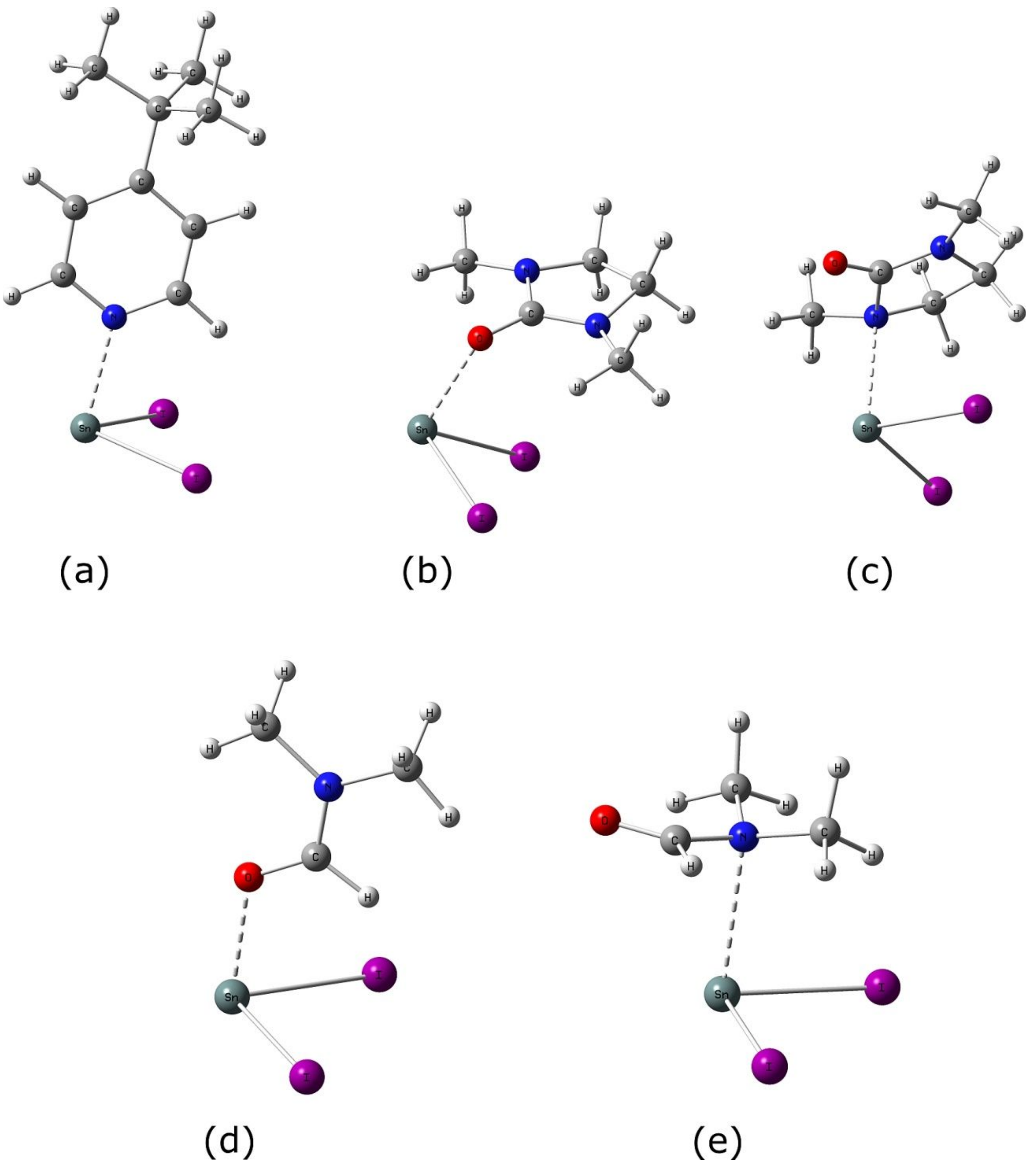

Figure S3. The geometries optimized in DMF solution for (a) SnI_2:TBP, (b) SnI_2:DMI formed via the Sn←O coordination bond, (c) SnI_2:DMI via Sn←N, (d) SnI_2:DMF via Sn←O, and (e) SnI_2:DMI via Sn←N. The colors coding individual elements are the same for all four complexes. The Sn←donor coordination bonds are denoted with a dashed line.

Complexes of 1:2 form

At the second stage of the computational part of the study, a series of complexes in 1:2 stoichiometry were examined. A great number of their initial geometries were optimized to identify their preferred isomers. For each 1:2 complex, at least two isomers were found and the isomer of the lowest energy was designated as the preferred one. For example, the isomers found for SnI_2:2TBP, SnI_2:2DMI and SnI_2:2DMF in DMF solution are listed in Tables S3–S5. Geometrical and energetic parameters of the

preferred isomers are summarized in Table S6. Figure S2 shows the preferred isomers of 1:2 complexes in DMF solution to exemplify the calculated structures.

The preferred isomers of SnI_2:2DMI and SnI_2:2DMF show the Sn←O coordination bonds with the DMI and DMF molecules occupying the axial positions of the coordination polyhedra. Such isomers are preferred both in solutions and in the gas phase. The Sn center of SnI_2 in the gas phase also tends to coordinate two TBP molecules at the axial positions. However, the preferred isomer of SnI_2:2TBP in DMF and DMSO solutions demonstrates two equatorial Sn←N bonds.

It is evident from the $E_{complex}$ values of three 1:2 complexes (Table S6) that the formation of SnI_2:2TBP in DMF and DMSO solutions is most energetically favorable. In general, the trends in the $E_{complex}$ energies of 1:1 complexes are still valid for the 1:2 complexes. The E_{int} values of SnI_2:2TBP in the solutions indicate that the introduction of the second TBP molecule into the complex strengthens each SnI_2-TBP pair interaction. The reverse is observed for SnI_2:2DMI and SnI_2:2DMF, as well as for the gas-phase SnI_2:2TBP complex. Such a behavior seems to result from the isomeric preference of these complexes. The values of E_{int} essentially follow the trend in $E_{int,tot}$, proving that the pairwise SnI_2-solvent interactions are dominant for the 1:2 complexes. It is worth noting that the $E_{int,tot}$ energy of 1:2 complexes includes the doubled E_{int} energy and, additionally, the contributions from solvent-solvent and three-body interactions.

Table S4. Selected parameters of the isomers found for SnI_2:2TBP in DMF solution. [a]

Isomer [b]	Parameter		
	d(Sn←donor)	*a*(I–Sn–I)	$E_{complex}$
N_{eq}→Sn←N_{eq}	232.8	167.3	-163.5
N_{ax}→Sn←N_{ax}	255.2	97.5	-158.7

[a] Distance (*d*) is given in pm, angle (*a*) in degrees, energy ($E_{complex}$) in kJ mol^{-1}.

[b] The equatorial and axial positions occupied by the TBP molecules in the complex are denoted by N_{eq} and N_{ax}, respectively.

Table S5. Selected parameters of the isomers found for SnI_2:2DMI in DMF solution. [a]

Isomer [b]	Parameter		
	d(Sn←donor)	*a*(I–Sn–I)	$E_{complex}$
O_{ax}→Sn←O_{ax}	247.2	94.7	-147.5
O_{eq}→Sn←O_{eq}	223.0	164.3	-133.6

[a] Distance (*d*) is given in pm, angle (*a*) in degrees, energy ($E_{complex}$) in kJ mol^{-1}.

[b] The equatorial and axial positions occupied by the DMI molecules in the complex are denoted by the subscripts 'eq' and 'ax', respectively.

Table S6. Selected parameters of the isomers found for SnI_2:2DMF in DMF solution. [a]

Isomer [b]	Parameter		
	d(Sn←donor)	*a*(I–Sn–I)	$E_{complex}$
O_{ax}→Sn←O_{ax}	246.5	95.4	-128.0
O_{ax}→Sn←N_{ax}	227.8;316.4	96.0	-116.2
N_{ax}→Sn←N_{ax}	282.5;283.4	97.1	-86.7
O_{ax}→Sn←N_{eq}	250.7;272.6	92.5	-79.7
N_{ax}→Sn←N_{eq}	339.1;284.2	96.1	-76.8

[a] Distance (*d*) is given in pm, angle (*a*) in degrees, energy ($E_{complex}$) in kJ mol^{-1}.

[b] The equatorial and axial positions occupied by the DMF molecules in the complex are denoted by the subscripts 'eq' and 'ax', respectively.

Table S7. Geometrical and energetic parameters for three 1:2 complexes in their preferred isomers in DMF solution. Values of the parameters for the complexes in DMSO solution and in gas phase are given in parentheses and square brackets, respectively. [a]

Parameter	SnI_2:2TBP	SnI_2:2DMI	SnI_2:2DMF
d(Sn←donor)	232.8<br>(232.7)<br>[257.4]	247.2<br>(247.3)<br>[249.4]	246.5<br>(246.7)<br>[249.3]
d(Sn–I)	313.4;314.6<br>(313.4;314.6)<br>[285.6]	292.2<br>(292.2)<br>[289.1]	292.4<br>(292.4)<br>[289.6]
a(I–Sn–I)	167.3<br>(167.1)<br>[99.0]	94.7<br>(95.2)<br>[97.8]	95.4<br>(95.3)<br>[95.8]
$E_{complex}$	-163.5<br>(-165.7)<br>[-170.3]	-147.5<br>(-148.3)<br>[-183.3]	-128.0<br>(-128.1)<br>[-155.2]
$E_{int,tot}$ [b]	-298.2<br>(-321.0)<br>[-183.9]	-184.8<br>(-204.2)<br>[-223.4]	-189.8<br>(-205.6)<br>[-188.0]
E_{int} [b]	-156.3<br>(-167.8)<br>[-110.1]	-114.4<br>(-123.7)<br>[-127.8]	-115.8<br>(-123.5)<br>[-107.5]

$E_{int,tot}$ [c]	-283.3<br>(-307.5)<br>[-164.5]	-147.5<br>(-162.9)<br>[-182.2]	-173.4<br>(-185.6)<br>[-164.5]
E_{int} [c]	-149.5<br>(-161.2)<br>[-102.0]	-97.5<br>(-104.1)<br>[-110.5]	-109.9<br>(-115.8)<br>[-99.4]

[a] Distances (*d*) are given in pm, angles (*a*) in degrees, energies ($E_{complex}$, $E_{int,tot}$) in kJ mol^{-1}.

[b] Energies calculated at the BP-D/aug-cc-pVTZ(-PP) level of theory.

[c] Energies calculated at the SCS-MP2/aug-cc-pVTZ(-PP) level of theory.

Hall Effect

We used AC magnetic field with a lock-in amplifier to enhance a low Hall effect signal due to low mobility and high conductivity values. 4-probe Hall effect and mobility were measured on encapsulated $FASnI_3$ samples in He environment at room temperature. We used 0.6 T magnetic field amplitude and a frequency 100 Hz. We do not observe any significant resistivity variation before ($2.5\cdot10^4$ Ω) and after ($2.49\cdot10^4$ Ω) measurements; thus, the ionic conductivity can be neglected.

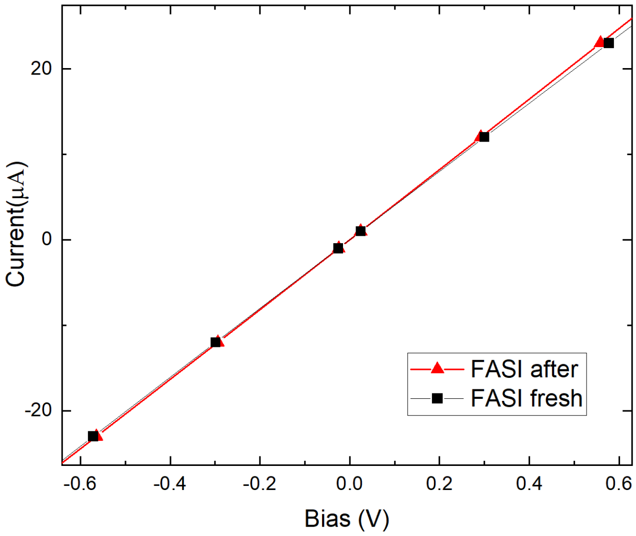

Figure S4. Current bias measurements on TBP samples before and after light exposure.

Table S8. Charge transport properties characterised by Hall effect and conductivity

**Sample**	**Mobility, $cm^2V^{-1}s^{-1}$**	**Concentration, p, cm^{-3}**	**Conductivity 4 probe, $(\Omega\cdot cm)^{-1}$**	**Conductivity 2 probe, $(\Omega\cdot cm)^{-1}$**	**Electron traps, cm^{-3}**
ref	0.04	$1.9\cdot10^{19}$	$8\cdot10^{-2}$	$7\cdot10^{-2}$	$2\cdot10^{19}$
TBP new	0.083	$1.1\cdot10^{18}$	$3.3\cdot10^{-3}$	$6.1\cdot10^{-3}$	$0.9\cdot10^{18}$

The charge transport model involving non-radiative[2], radiative, and Auger recombination channels is described by the following equations:

$$\frac{dp}{dt} = 0 = -C_b(pn - n_i^2) - B_p(np^2 - n_0p_0^2) - p\sigma_h v_h n_t + G \qquad (1)$$

Where G is generation rate; C_b and B_p are radiative and Auger constants; n_t and σ_h are trap density and capture crosssection, n_0 and p_0 are dark carrier concentrations.

Table S9. Constants used in charge simulationc

B_p cm^3s^{-1}	C_b cm^3s^{-1}	σ_h cm^2	v_e cms^{-1}	v_h cms^{-1}	E_g, eV
5×10^{-29}	5×10^{-10}	3×10^{-17}	3×10^{7}	3×10^{7}	1.8

Stability

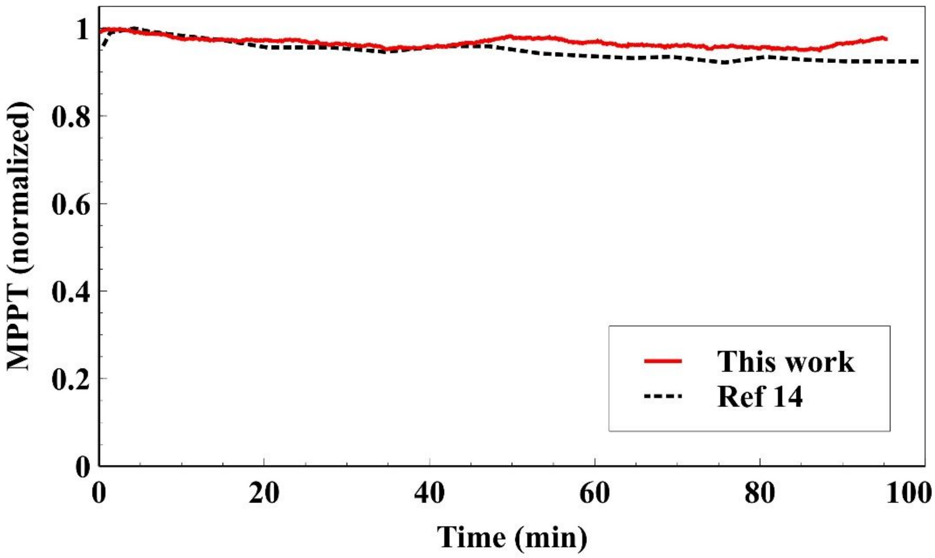

Figure S5: MPPT of TBP-treated THP device under AM1.5 illumination.

Finally, the devices obtained using TBP have been tested with a maximum power point tracking (MPPT) routine to verify the PCE stability under operation (Figure). The devices retained their initial PCE (6%) for 100 minutes of MPPT characterization under 1.5M illumination. The stability of these devices can be related to two reasons: the adoption of a DMSO-free process which prevents the oxidation of Sn^{2+} to Sn^{4+},, reducing the number of lattice defects and therefore reducing the migration of ions under illumination; and the formation of more compact crystals, aided by TBP. In Figure is also reported, as comparison, the stability test under MPPT extracted from Ref 18.

Movie S1: crystallization kinetic of tin perovskite thin film without the addition of tBP

Movie S2: crystallization kinetic of tin perovskite thin film with the addition of tBP

References

1. Becke AD (1988) Density-functional exchange-energy approximation with correct asymptotic behavior. Phys Rev A 38:3098–3100

2. Perdew JP (1986) Density-functional approximation for the correlation energy of the inhomogeneous electron gas. Phys Rev B 33:8822–8824

3. Grimme S, Ehrlich S, Goerigk L (2011) Effect of the damping function in dispersion corrected density functional theory. J Comput Chem 32:1456–1465

4. Peterson KA (2003) Systematically convergent basis sets with relativistic pseudopotentials. I. Correlation consistent basis sets for the post-d group 13–15 elements. J Chem Phys 119:11099–11112

5. Dunning TH, Jr. (1989) Gaussian basis sets for use in correlated molecular calculations. I. The atoms boron through neon and hydrogen. J Chem Phys 90:1007–1023

6. Matczak P (2019) N→Sn coordination in the complexes of tin halides with pyridine: a comparison between Sn(II) and Sn(IV). Appl Organometal Chem 33:e4811

7. Bankiewicz B, Kupfer S, Matczak P (2021) Tuning the metal–ligand bond in the σ-complexes of stannylenes and azabenzenes. J Comput Chem 42:2103–2115

8. Marenich AV, Cramer CJ, Truhlar DG (2009) Universal solvation model based on solute electron density and a continuum model of the solvent defined by the bulk dielectric constant and atomic surface tensions. J Phys Chem B 113:6378–6396

9. Grimme S (2003) Improved second-order Møller-Plesset perturbation theory by separate scaling of parallel- and antiparallel-spin pair correlation energies. J Chem Phys 118:9095–9102

10. Matczak P, Wojtulewski S (2015) Performance of Møller-Plesset second-order perturbation theory and density functional theory in predicting the interaction between stannylenes and aromatic molecules. J Mol Model 21:41

11. Boys SF, Bernardi F (1970) The calculation of small molecular interactions by the differences of separate total energies. Some procedures with reduced errors. Mol Phys 19:553–566

12. Frisch MJ, Trucks GW, Schlegel HB, Scuseria GE, Robb MA, Cheeseman JR, Scalmani G, Barone V, Petersson GA, Nakatsuji H, Li X, Caricato M, Marenich AV, Bloino J, Janesko BG, Gomperts R, Mennucci B, Hratchian HP, Ortiz JV, Izmaylov AF, Sonnenberg JL, Williams-Young D, Ding F, Lipparini F, Egidi F, Goings J, Peng B, Petrone A, Henderson T, Ranasinghe D, Zakrzewski VG, Gao J, Rega N, Zheng G, Liang W, Hada M, Ehara M, Toyota K, Fukuda R, Hasegawa J, Ishida M, Nakajima T, Honda Y, Kitao O, Nakai H, Vreven T, Throssell K, Montgomery JA, Jr., Peralta JE, Ogliaro F, Bearpark MJ, Heyd JJ, Brothers EN, Kudin KN, Staroverov VN, Keith TA, Kobayashi R, Normand J,

Raghavachari K, Rendell AP, Burant JC, Iyengar SS, Tomasi J, Cossi M, Millam JM, Klene M, Adamo C, Cammi R, Ochterski JW, Martin RL, Morokuma K, Farkas O, Foresman JB, Fox DJ (2016) Gaussian 16, Rev. C.01. Gaussian, Inc., Wallingford CT

13. Broeckaert L, Geerlings P, Růžička A, Willem R, De Proft F (2012) Can aromatic π-clouds complex divalent germanium and tin compounds? A DFT study. Organometallics 31:1605–1617

14. Aitken CT, Onyszchuk M (1985) Preparation and spectroscopic studies of some cyclic urea adducts of triphenyl-tin and -lead halides. J Organomet Chem 295:149–158

15. Maercz M, Wragg DS, Dietzelc PDC, Fjellvag H (2013) Poly[bis(1,3-dimethylimidazolidin-2-one)(l2-2,5-dioxidoterephthalato)-zirconium(IV)]. Acta Cryst E 69:m152

16. Yamamoto S, Mitsuhashi R, Mikuriya M, Koikawa M (2021) Crystal structure, magnetic properties, and structural prediction for an oxidovanadium(IV) complex $[VO(dmf)_5][PF_6]_2$. J Coord Chem 74:1222–1232

17. De O. Santiago PH, De A. Duarte E, Nascimento ECM, Martins JBL, Castro MS (2022) A binuclear copper(II) complex based on hydrazone ligand: Characterization, molecular docking, and theoretical and antimicrobial investigation. Appl Organomet Chem 36:e6461

18. Jiang, X. *et al.* One-Step Synthesis of SnI2·(DMSO)x Adducts for High-Performance Tin Perovskite Solar Cells. *J. Am. Chem. Soc.* **143**, 10970–10976 (2021)
